# A novel compound targets the feline infectious peritonitis virus nucleocapsid protein and inhibits viral replication in cell culture

**DOI:** 10.1016/j.jbc.2023.102976

**Published:** 2023-02-03

**Authors:** Nazleen Mohseni, Austin Royster, Songyang Ren, Yutian Ma, Melissa Pintado, Mohammad Mir, Sheema Mir

**Affiliations:** College of Veterinary Medicine, Western University of Health Sciences, Pomona, California, USA

**Keywords:** virus, nucleocapsid protein, antivirals, coronavirus, virus replication, 5′ NCR, 5′ noncoding region, bis-ANS, 4,4′-dianilino-1,1′-binaphthyl-5,5′-disulfonic acid, dipotassium salt, BLI, biolayer interferometry, CRFK, Crandell Rees Feline Kidney, DMEM, Dulbecco's modified Eagle's medium, DMSO, dimethyl sulfoxide, FBS, fetal bovine serum, FCoV, feline alphacoronavirus, FECV, feline enteric coronavirus, FIP, feline infectious peritonitis, FIPV, feline infectious peritonitis virus, HCoV-OC43, human coronaviruses subtype OC43, HUVEC, human umbilical vein endothelial cell, MERS, Middle East respiratory syndrome, MOI, multiplicity of infection, ORF, open reading frame, SARS-CoV-2, severe acute respiratory syndrome coronavirus-2

## Abstract

Feline infectious peritonitis (FIP) is a serious viral illness in cats, caused by feline coronavirus. Once a cat develops clinical FIP, the prognosis is poor. The effective treatment strategy for coronavirus infections with immunopathological complications such as SARS-CoV-2, MERS, and FIP is focused on antiviral and immunomodulatory agents to inhibit virus replication and enhance the protective immune response. In this article we report the binding and conformational alteration of feline alphacoronavirus (FCoV) nucleocapsid protein by a novel compound K31. K31 noncompetitively inhibited the interaction between the purified nucleocapsid protein and the synthetic 5′ terminus of viral genomic RNA *in vitro*. K31 was well tolerated by cells and inhibited FCoV replication in cell culture with a selective index of 115. A single dose of K31inhibited FCoV replication to an undetectable level in 24 h post treatment. K31 did not affect the virus entry to the host cell but inhibited the postentry steps of virus replication. The nucleocapsid protein forms ribonucleocapsid in association with the viral genomic RNA that serves as a template for transcription and replication of the viral genome. Our results show that K31 treatment disrupted the structural integrity of ribonucleocapsid in virus-infected cells. After the COVID-19 pandemic, most of the antiviral drug development strategies have focused on RdRp and proteases encoded by the viral genome. Our results have shown that nucleocapsid protein is a druggable target for anticoronavirus drug discovery.

Coronaviruses, classified into four genera, alpha-, beta-, gamma- and delta-coronaviruses, belong to a large family of RNA viruses that infect a wide variety of mammalian and avian hosts, causing a broad spectrum of diseases. Recent emergence and transmission of deadly coronaviruses such as severe acute respiratory syndrome coronavirus-2 (SARS-CoV-2) and Middle East respiratory syndrome Coronavirus (MERS-CoV) has raised awareness about the risk of coronavirus infection to human populations by increasing the contact between humans and animals infected with coronaviruses. Feline alphacoronavirus (FCoV) infects cats worldwide at a very young age. In the United Kingdom around 40% of the domestic cats are seropositive for FCoV (1). The infection rates can go as high as 90% in multicat households (2). FCoV infection is mostly asymptomatic or can result in a mild, self-resolving gastrointestinal illness. When the illness remains limited to the gastrointestinal tract, the virus is commonly referred to as feline enteric coronavirus (FECV). In about 5% of infected cats the virus infects white blood cells (macrophages and monocytes) and disseminates to entire body causing a multisystemic, immune-mediated life-threatening disease commonly known as feline infectious peritonitis (FIP), and the virus is referred to as FIP virus (FIPV) (3). A widely accepted hypothesis suggests that FECV undergoes mutations that give rise to more virulent FIPV. However, the role of genetic differences between FECV and FIPV in the differential pathogenicity remains to be elucidated (3, 4). Once a cat develops clinical FIP, the prognosis worsens, and the highly progressive disease almost always becomes fatal.

FIP occurs in two major forms, effusive (wet) form or noneffusive (dry) form. The more common wet form of FIP is characterized by accumulation of fluids in the abdominal and/or chest cavities. The characteristic exudates found in the body cavities of animals with wet FIP is rich in proteins and macrophages with high viral load (5). The characteristic immunopathogenesis and lymphopenia in FCoV-infected cats are also observed in humans infected with other coronaviruses such as SARS-CoV-2 and MERS. The similarity in virion structure and molecular mechanism of pathogenesis among coronaviruses has provided insights for the use of FCoV inhibitor (GC376) in the treatment of SARS-CoV-2 (6, 7). The inhibitor GC376 targets FCoV 3CL^pro^ (an essential protease) and inhibits the replication of both FCoV and SARS-CoV-2 (6). Another effective and widely used drug for the treatment of FIP is a nucleoside analogue GS441524 with an optimum dosage of 4.0 mg/kg for at least 12 weeks (8). GS441524 interferes in the replication process of FCoV. The effective treatment strategy for the coronavirus infections with immunopathological complications such as SARS-CoV-2, MERS, and FIP is focused on using antiviral agents to inhibit the virus replication and immunomodulatory agents to enhance the protective immunity and decrease the pathological immune responses in the host. Polyprenyl immunostimulant, a United States Department of Agriculture–licensed substance to treat feline rhinotracheitis is used off-label to treat FIP in the United States (9). Remdesivir, approved by the US Food and Drug Administration to treat COVID-19, is being used by veterinarians in the United Kingdom and Australia to treat FIP with a high success rate. Since COVID-19 pandemic the anticoronavirus drug development initiatives have mainly focused on the discovery of anti-SARS-CoV-2 agents. A majority of anticoronavirus drug development approaches target the coronavirus encode surface protein, the protease, the helicase, and the replication complex (10). This article reports a novel compound that targets the FCoV nucleocapsid protein and inhibits FCoV replication with high selective index. The compound also inhibits the replication of human coronaviruses (HCoV) subtype OC43 (HCoV-OC43), a beta-coronavirus that causes severe lower respiratory tract illness, especially in immunocompromised humans (11). The suspected animal-to-human spread of four beta-coronaviruses, including the HCoV-OC43 (1890), SARS-CoV-1 (2003), MERS-CoV (2012), and SARS-CoV-2 (2019), proposes their high potential to cause pandemics (12).

The positive-sense viral genome of FCoV is composed of 29,125 nucleotides, excluding the 3′ poly(A) tail (13). The viral genome has six open reading frames (ORFs). ORF1a (nt 312–12,208) and ORF1b (nt 12,164–20,209) encode the nonstructural proteins (13). The remaining four ORFs encode the spike glycoprotein (160 kDa), the envelope protein (9.4 kDa), the membrane protein (29.8 kDa), and the nucleocapsid protein (42.7 kDa) (13). The viral genome is encapsidated by the nucleocapsid protein (N) and packaged in the lipid envelop harboring the spike, envelop, and membrane structural proteins.

The coronavirus N is highly conserved and plays diverse roles in the coronavirus replication cycle. The X-ray crystallographic studies showed that coronavirus N has highly conserved structural architecture, composed of N-terminal (14, 15) and C-terminal (16, 17) RNA-binding domains separated by a disordered linker region. The primary and well-understood role of N is to selectively encapsidate the viral genome to generate ribonucleocapsids. The ribonucleocapsids serve as efficient templates for transcription and replication of the viral genome by the viral RdRp (18). X-ray crystallographic studies have shown that the linker region of N directly interacts with the nonstructural protein (nsp3), a component of the viral replication/transcription complex, and the interaction plays a role in the virus replication (19, 20, 21). The viral nucleocapsids are specifically incorporated into new virions by the highly specific interaction between the C-terminal domain of the viral envelop M protein and the C-terminal domain of the N engaged in nucleocapsids (22). The coronavirus N has RNA chaperon activity, required for template switching during transcription/replication of the viral genome (23, 24). Coronavirus N modulates the host cell cycle by regulating the cyclin-CDK activity that leads to an arrest in the progression of S-phase, allowing the uninterrupted synthesis of viral components (25). The high pathogenicity of severe acute respiratory coronavirus (SARS CoV) has been attributed to the inhibition of type I interferon response by the SARS CoV N (26). Coronavirus N has been reported to cause the host translation shutoff by directly binding to the translation elongation factor 1α, making the host translation machinery abundantly available for the synthesis of viral proteins (27). These important functions of N clearly demonstrate that multifunctional N is a novel target for therapeutic intervention of coronaviruses.

In this article, we demonstrate the inhibition of FCoV replication by a novel molecule, [4-(3-Bromophenyl)-3a,4,5,9b-tetrahydro-3H-cycloppenta [c] quinoline-6-carboxylix acid], referred from here on as K31. K31 has been previously reported to inhibit Andes virus, a new world hantavirus that causes hantavirus cardiopulmonary syndrome with a mortality rate of 50% in humans (28, 29). The old-world hantaviruses cause hemorrhagic fever with renal syndrome, with a mortality rate of 15% in humans. Unlike coronaviruses, hantaviruses have trisegmented negative-sense RNA genome that encodes nucleocapsid protein, RdRp, and two glycoproteins Gn and Gc. Similar to coronaviruses, the hantavirus nucleocapsid protein encapsidates the viral genome and forms ribonucleocapsids that serve as templates for viral mRNA synthesis and replication of the viral genome. We demonstrate that K31 binds to FCoV N and inhibits virus replication in cell culture model with a selective index of 115. Our results suggest that K31 can be further developed as antiviral therapeutic for the treatment of FIP.

## Results

### Expression and purification of FCoV N in bacteria

**Figure 1:**
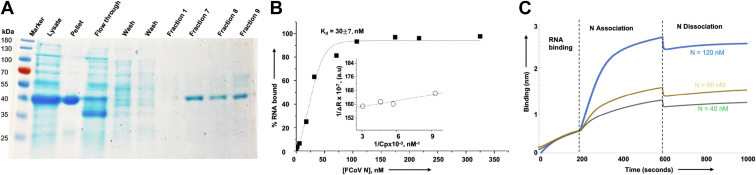
**Purification of FIPV N protein (N) and RNA binding.***A*, purification of FCoV nucleocapsid protein (N) using Ni-NTA chromatography. *B*, binding profile, generated by filter binding analysis, for the interaction of N with the vRNA 5′ NCR sequence (5′ACUUUUAAAGUAAAGUGAGUGUAGCGUGGCUAUAAC3′) in binding buffer containing 160 mM NaCl concentration. Inset shows the double reciprocal plot for the calculation of ΔR_max_, used to calculate the percent bound RNA at each input concentration of N (see Experimental procedures for details). *C*, representative biolayer interferometry sensograms showing the overtime association and dissociation of N with the vRNA 5′ NCR sequence shown above. The sensograms were generated at three different concentrations of N. For details see Experimental procedures.

The gene encoding the FCoV N (strain 79-1146) (GenBank: AY994055.1) was cloned between Nde1 and HindIII restriction sites in pet30a(+) plasmid and expressed in *Escherichia coli* as C-terminally His-tagged fusion protein (see Experimental procedures for details). The protein was purified by Ni-NTA chromatography on AKT Pure protein purification system (GE Healthcare) using the native purification method, as reported (30). As shown in Figure 1*A*, the purified N in fraction 7 was free of detectable heterologous bacterial proteins and its electrophoretic mobility was consistent with its expected molecular mass of ∼42.6 KDa.

### Purified N binds to the vRNA 5′ noncoding region with specificity

To determine whether the purified N is biologically active, we examined its RNA binding activity using the filter binding analysis, as mentioned in Experimental procedures. Briefly, 36 nucleotides from the 5′ terminus of viral genomic RNA were synthesized and radiolabeled with α-^32^P GTP, as reported (31, 32, 33) (see Experimental procedures for details). A fixed concentration of radiolabeled RNA was incubated with increasing concentrations of purified N, and the resulting mixture was filtered through nitrocellulose filter. The radioactive signal retained on the filter at each input concentration of N was used to generate the binding profile for the calculation of dissociation constant (K_d_), as discussed in Experimental procedures. The hyperbolic nature of the binding profile (Fig. 1*B*) demonstrates the independent binding between N and viral 5′ noncoding region (5′ NCR). Analysis of the binding data revealed that N bound to the radiolabeled 5′ NCR with a K_d_ value of ∼30 nM. Using the same approach, we studied the binding between N and radiolabeled viral NCR at increasing salt concentrations to examine the specificity of N–5′ NCR interactions. As demonstrated in Table 1, we did not observe a noticeable change in the K_d_ values at increasing salt concentrations. This clearly demonstrates that N binds to the viral NCR sequence with high specificity.

**Table 1:** Filter binding analysis for the interaction of FCoV N with the radiolabeled 5′ NCR sequence of the viral genome

NaCl concentration	RNA^a^	Protein	K_d_ ± SD^b^( nM)
80 mM	FCoV 5′ NCR	FCoV N	29.0 ± 1.6
160 mM	FCoV 5′ NCR	FCoV N	30.7 ± 7.5
320 mM	FCoV 5′ NCR	FCoV N	35.0 ± 7.0

a RNA (5′ NCR = 5′ACUUUUAAAGUAAAGUGAGUGUAGCGUGGCUAUAAC3′).

b K_d_ = dissociation constant, was calculated as mentioned in Experimental procedures. The K_d_ values calculated from three independent experiments were averaged, and the mean K_d_ values are shown in the table. SD represents the standard deviation.

We next used biolayer interferometry (BLI) to further confirm the results of filter binding analysis, as mentioned in Experimental procedures. Briefly, 36 nucleotides from the 5′ terminus of viral genomic RNA were synthesized and biotinylated, as reported (31, 32, 33). The biotinylated 5′ NCR sequence was immobilized on the streptavidin biosensor, followed by the examination of association and dissociation kinetics of purified N at different concentrations (Fig. 1*C*). Analysis of the kinetic data revealed that N bound to the 5′ NCR sequence with a very fast on rate and a very slow off rate, generating a dissociation constant K_d_ ∼ 18 nM (Table 2). The interaction between N and viral 5′ NCR sequence, confirmed by two independent experimental approaches, clearly demonstrates that the purified N is biologically active.

**Table 2:** Biolayer interferometry to study the interaction of N with 5′ NCR and K31

Interactors	K_d_ ± SD (nM)	K_ass_ (M-1S-1)	K_dis_ (S-1)
N + 5′ NCR	18 ± 1.8	3.195 × 10^5^	5.931 × 10^−3^
N + K31	350 ± 17	4.292 × 10^1^	2.763 × 10^−5^

K_d_ = K_dis_/K_ass._ SD ∼5 to 10% of the K_d_ value.

### K31 inhibits N–RNA interaction *in vitro*

**Figure 2:**
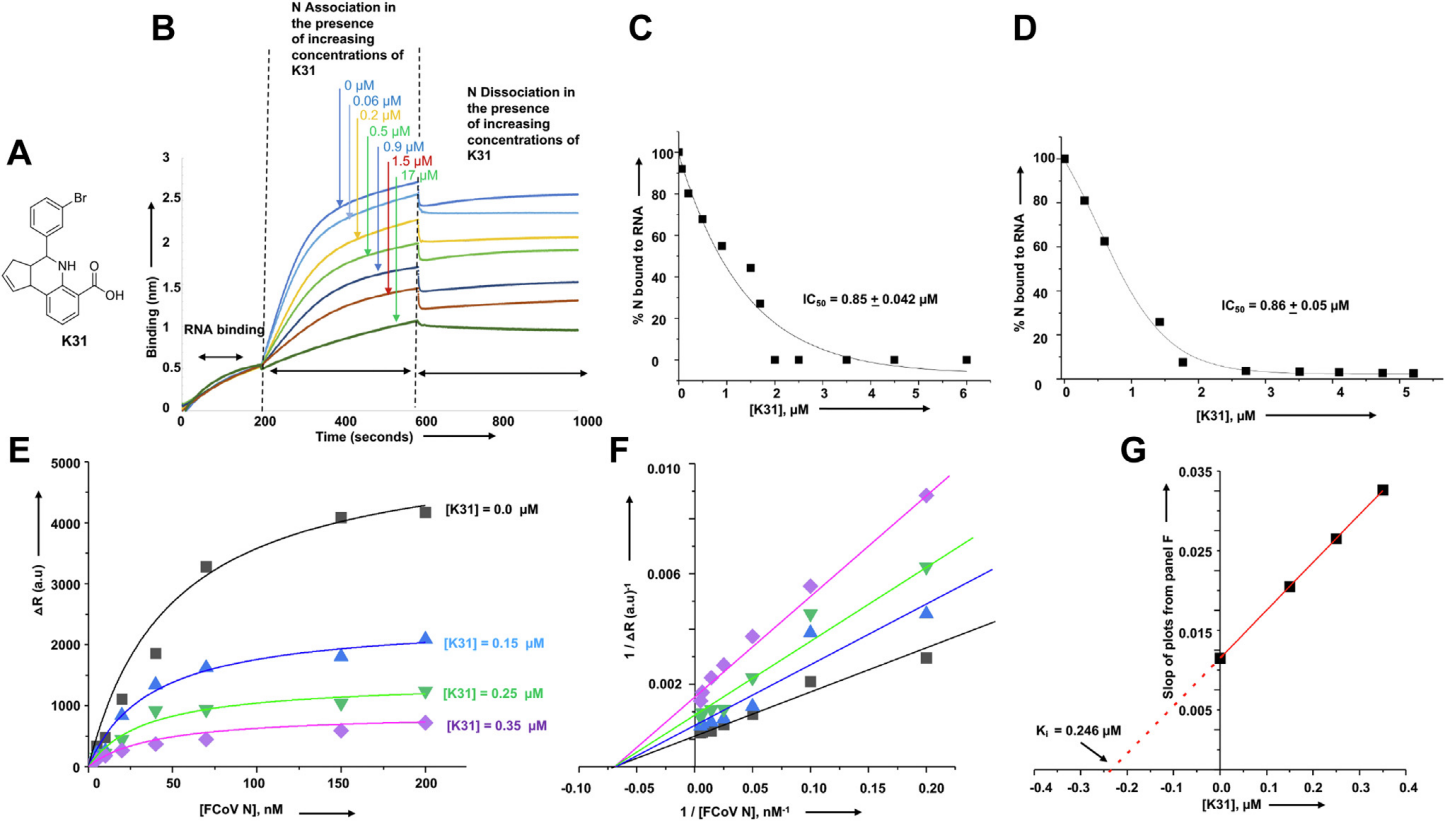
**Inhibition of N–vRNA interaction by K31.***A*, structure of K31. *B*, representative biolayer interferometry sensograms showing the overtime association and dissociation of N (120 nM) with the vRNA 5′ NCR sequence in the presence of increasing input concentration of K31, shown by *vertical arrows*. *C*, the inhibition profile showing the percentage of N bound to the vRNA 5′ NCR sequence at each input concentration of K31. The data from A were used to calculate the percentage of N bound to the vRNA 5′ NCR sequence at each input concentration of K31, as mentioned in Experimental procedures. *D*, the N–5′ NCR complex was chased with increasing concentrations of K31. The percentage of N bound to the RNA was plotted *versus* each input concentration of K31 to generate the inhibition profile for the calculation of IC_50_. *E*, binding profiles for N–5′ NCR interaction, generated by plotting ΔR (differential radioactive signal) *versus* N concentration. The four binding profiles were generated at four different K31 concentrations as shown (see Experimental procedures for details). *F*, the Lineweaver–Burk plots were generated using the data from *E*. *G*, the secondary plot was generated by plotting the slopes of Lineweaver–Burk plots from F *versus* input K31 concentration. The data points were fit to straight line.

Previously a high-throughput screening approach identified K31(Fig. 2*A*) as a novel inhibitor that inhibited hantavirus replication in cell culture (28, 29). We wanted to determine whether K31 inhibits the interaction between FCoV N and vRNA 5′ NCR. Biolayer interferometry was used to monitor the impact of K31 upon the N–RNA interaction *in vitro*, as mentioned in Experimental procedures. Association and dissociation kinetics of purified N with the immobilized synthetic vRNA 5′ NCR was studied in the presence of increasing concentrations of K31. As shown in Figure 2*B*, K31 inhibited the association of N with the vRNA 5′ NCR in a dose-dependent manner. Analysis of the inhibition data (Fig. 2*C*) revealed that K31 inhibited N–NCR interaction with an inhibition constant (IC_50_) of ∼0.85 μM.

To confirm these results, we examined the impact of K31 upon the N–5′ NCR complex using filter binding analysis (see Experimental procedures). Briefly, a preformed complex between N and radiolabeled 5′ NCR was chased with increasing concentrations of K31. The intact N–5′ NCR complex was quantified by filter binding analysis and plotted *versus* the input concentration of K31 to generate the inhibition profile (Fig. 2*D*). Analysis of the filter binding data confirmed that K31 inhibited the N–5′ NCR interaction with an inhibition constant of ∼0.86 μM (Fig. 2*D*).

We next used filter binding analysis to delineate the mechanism of inhibition as mentioned in Experimental procedures. Briefly, N–5′ NCR complexes were formed by incubating a fixed concentration of radiolabeled 5′ NCR with increasing concentrations of N. The resulting complexes were further incubated with K31 at four different concentrations, followed by filtration through nitrocellulose filter. The radioactive signal retained on the filter was plotted *versus* N to generate four binding profiles corresponding to four K31 concentrations (Fig. 2*E*). The characteristic nature of Lineweaver–Burk plots (Fig. 2*F*), generated from binding data (Fig. 2*E*), demonstrates that K31 inhibits N–5′ NCR interaction in a noncompetitive manner (34, 35, 36). The secondary plot (Fig. 2*G*), generated by plotting the slops of Lineweaver–Burk plots *versus* K31 concentration demonstrated a K_i_ of ∼0.246 μM.

### K31 binds to FCoV N and induces a conformation change in it

**Figure 3:**
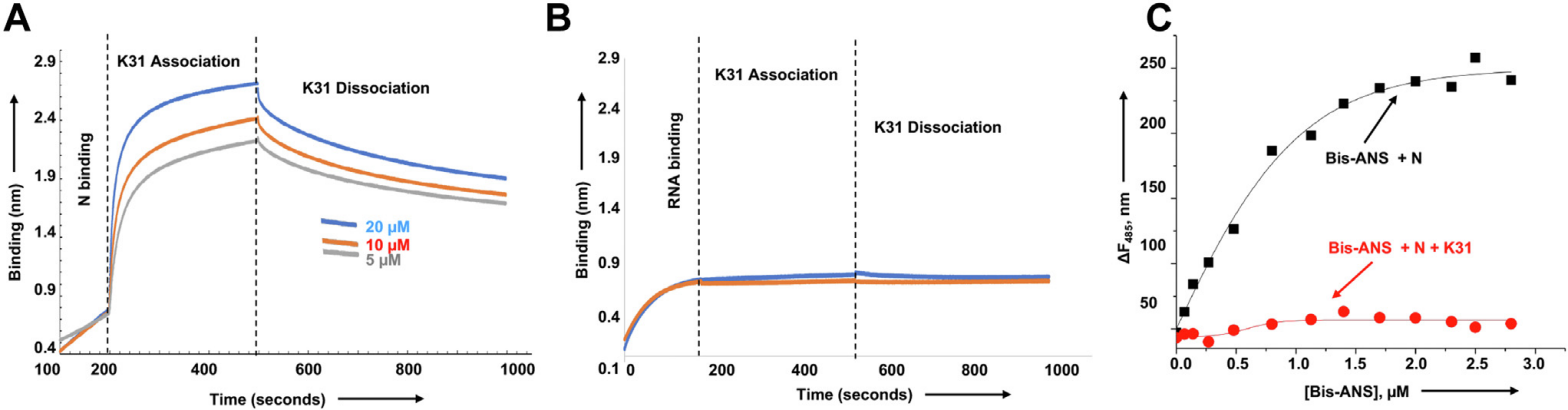
**Binding of N with K31.***A*, representative biolayer interferometry sensograms showing the overtime association and dissociation of K31 with the purified N. The sensograms were generated at three different concentrations (20 μM, 10 μM, and 5 μM) of K31, as shown by three different colors. For details see Experimental procedures. *B*, representative biolayer interferometry sensograms showing the overtime association and dissociation of K31 with the synthetic vRNA 5′ NCR sequence. The sensograms were generated at 20 μM and 10 μM of K31. No binding was observed. *C*, fluorescence titration of hydrophobic fluorophore (bis-ANS) with N, and N–K31 complex. The fluorophore was excited at 399 nm, and the fluorescence emission signal was recorded at 485 nm. Shown are the titration curves of bis-ANS binding with free N (*black filled square*) and N–K31 complex (*red circle*).

We wanted to determine whether the inhibition of N–5′ NCR interaction was due to the binding of K31 with the 5′ NCR sequence or FCoV N. Biolayer interferometry was used to monitor the potential binding of K31 to the FCoV N and synthetic 5′ NCR (see Experimental procedures). As shown in Figure 3*A*, K31 bound to FCoV N with different on and off rates (Table 1), generating a dissociation constant (k_d_) of ∼0.350 μM. Similar binding affinity (Ki ∼ 0.246 μM) was observed by Lineweaver–Burk plots (Fig. 2*G*). K31 did not show any interaction with the vRNA 5′ NCR (Fig. 3*B*).

We next wanted to determine whether K31 binding induces a conformational change in N. To do so we used 4,4′-dianilino-1,1′-binaphthyl-5,5′-disulfonic acid, dipotassium salt (bis-ANS) as probe to monitor the potential conformational alterations in N due to K31 binding, as reported (29). The fluorescence signal of bis-ANS increases upon binding to hydrophobic pockets of proteins and has been widely used to probe the conformational changes induced in proteins due to binding with diverse ligands (37, 38, 39, 40). It is evident from Figure 3*C* that the fluorescence signal of bis-ANS increases with higher magnitude after binding to free N as compared with the N–K31 complex. We did not observe a noticeable change in the fluorescence signal of bis-ANS after binding to the N–K31 complex (compare red and black lines in Fig. 3*C*). This suggests that binding of K31 reduces the number of available hydrophobic binding pockets for bis-ANS. It is likely that either K31 binding induces a conformational change that reduces the hydrophobic pockets in N or the K31-binding sites on N are themselves hydrophobic in nature.

### Cellular toxicity and inhibition of coronavirus replication

**Figure 4:**
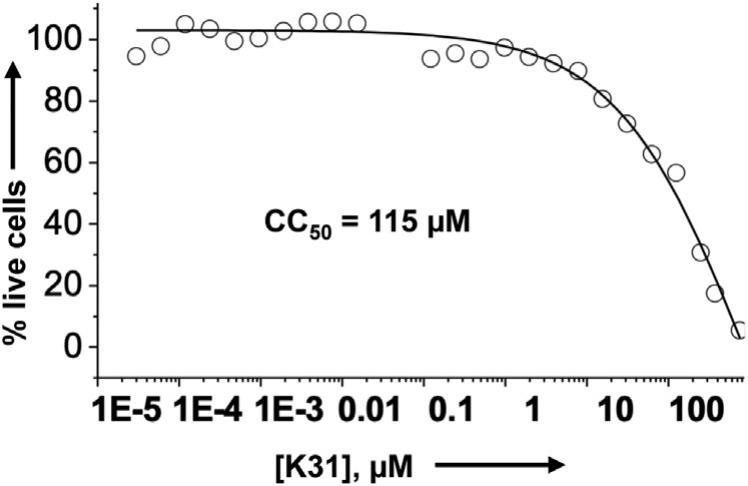
**Cytotoxicity of CRFK cells with K31.** CRFK cells were incubated with increasing concentrations of K31 for 48 h. Live cells at each input concentration of K31 were calculated as mentioned in Experimental procedures and plotted *versus* K31 concentration to generate the shown plot.

**Figure 5:**
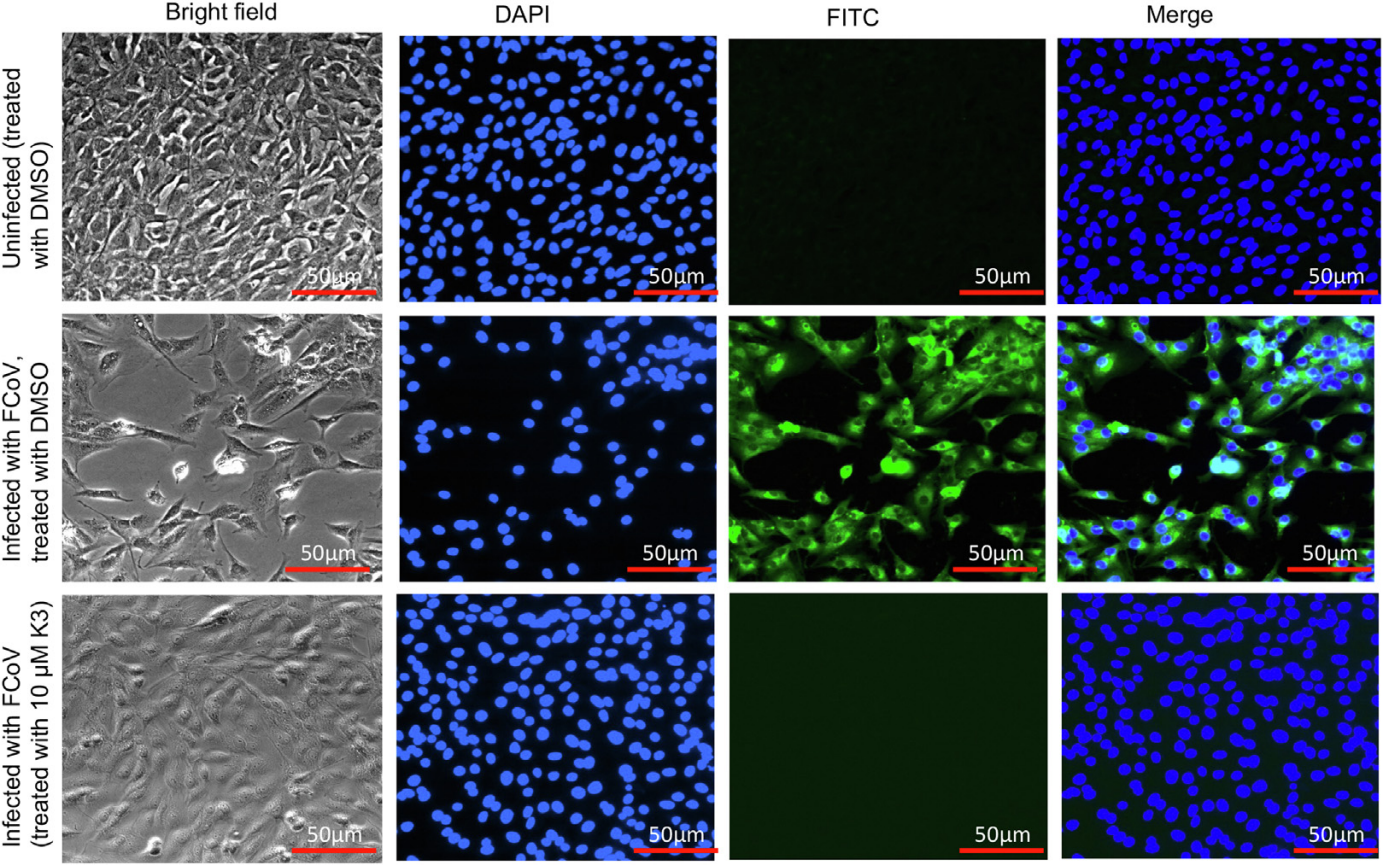
**Inhibition of FCoV by K31 in cell culture.** CRFK cells were grown in six-well plates and treated with either dimethyl sulfoxide (DMSO) or 10 μM K31 dissolved in DMSO. Immunostaining was carried out as mentioned in Experimental procedures.

Before examining the antiviral activity of K31 against FCoV virus in cell culture, we examined the cytotoxicity of K31 in FCoV virus permissive Crandell Rees Feline Kidney (CRFK) cells. The cytotoxicity of K31 in CRFK cells was examined as described in Experimental procedures. As shown in Figure 4, the CC_50_ (the concentration of K31 at which 50% cell death occurred) was 115 μM, suggesting a considerable tolerance of K31 by this cell line. Similar tolerance of K31 was previously reported in other cells lines including human umbilical vein endothelial cells (HUVECs), discussed later in this section (29). K31 was further assayed for antiviral activity. Briefly CRFK cells seeded in 24-well plates were infected with FCoV virus at an multiplicity of infection (MOI) of 0.1 and treated with either vehicle (dimethyl sulfoxide [DMSO]) or 10 μM K31 1 h post infection. Virus replication was examined by immunofluorescence staining 24 h post infection, as mentioned in Experimental procedures. As shown in Figure 5, the FCoV rapidly multiplied in untreated CRFK cells and induced cell death evident from rounding and detachment of cells from the plate surface (compare bright field images in Fig. 5). However, the treatment of infected cells with 10 μM K31 prevented both virus replication and virus-induced cell death. We did not notice FCoV replication in CRFK cells by immunofluorescence staining after K31 treatment for 24 h (compare FITC staining in K31-treated and untreated cells).

**Figure 6:**
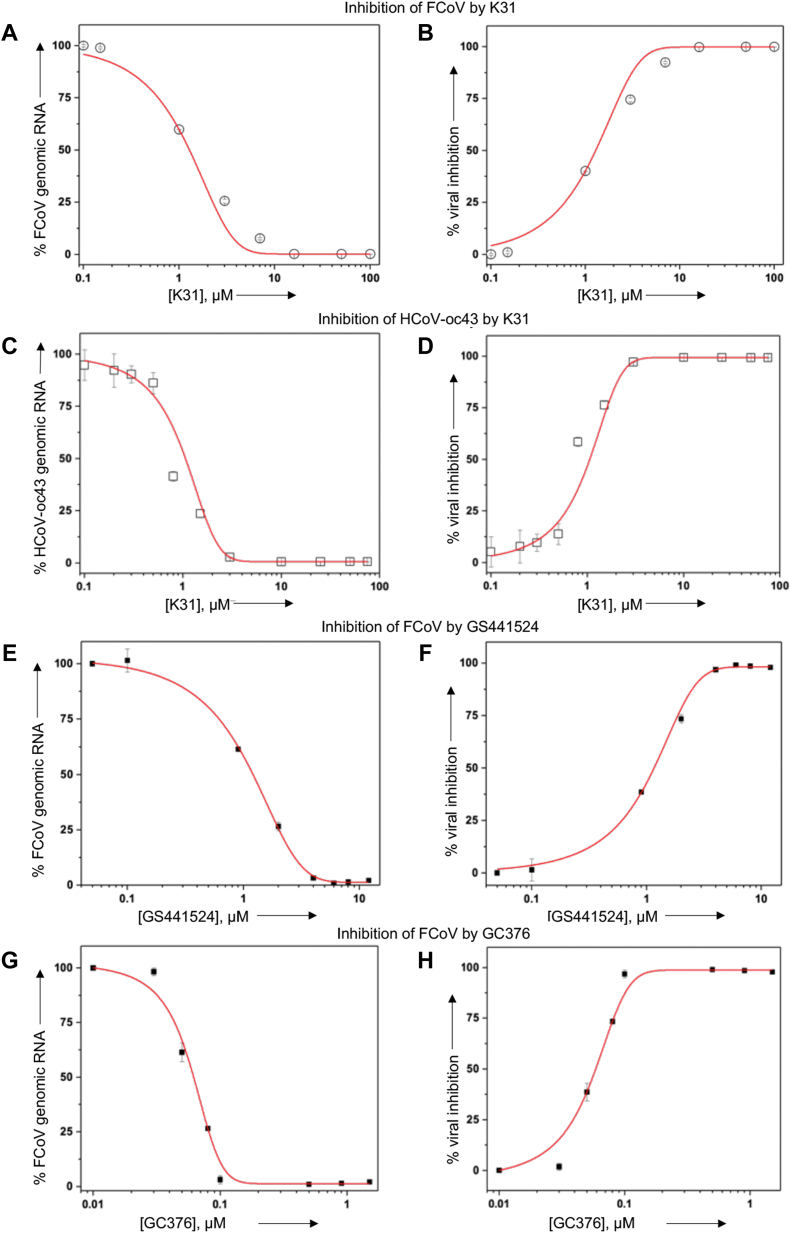
**Dose–response curve for the inhibition of FCoV and HCoV-OC43 by K31, GS441524, and GC376 in cell culture**. CRFK cells (*A*, *B* and *E*–*H*) and HUVECs (*C* and *D*) were infected with FCoV (strain FIPV-79-4418) and HCoV-OC43, respectively. Cells were incubated with increasing concentrations of K31 (*A*–*D*), GS441524 (*E* and *F*), and GC376 (*G* and *H*) for 24 h post infection. Cells were lysed, and viral genomic RNA was quantified at each input concentration of the inhibitor by real-time PCR. The percentage of viral genomic RNA related to untreated control was determined and plotted *versus* inhibitor concentration (*left panels*). The data from *left panels* were used to calculate percent viral inhibition by subtracting the *y*-axis value corresponding to each input inhibitor concentration from the *y*-axis value when inhibitor concentration was zero. The resulting percent viral inhibition was plotted *versus* the corresponding input inhibitor concentration (*right panels*). The data points were fit to dose–response curve using Origin 6.0 Pro for the calculation of EC_50_ values. See Experimental procedures for details.

To examine the dose–response impact of K31 upon FCoV replication, CRFK cells in 24-well plates were infected with FCoV at an MOI of ∼0.1 along with increasing concentrations of K31. The K31 was delivered to cells below the cytotoxicity range to ensure that viral inhibition observed was not due to cytotoxicity. Cells were washed 1 h post infection and incubated for additional 24 h with fresh medium containing K31. Virus replication was monitored by quantitative estimation of viral genomic RNA by real-time PCR, as mentioned in Experimental procedures. A plot of percent viral inhibition *versus* input concentration of K31 was used for the calculation of EC_50_, the concentration of the compound at which 50% virus replication was inhibited. As shown in Figure 6, *A* and *B*, K31 inhibited FCoV replication in cell culture with an EC_50_ of 1 ± 0.2 μM (Table 3). It is evident that K31 is highly potent with a selective index (SI) of 115 (SI = CC_50_/EC_50_). A similar strategy was used to determine whether K31 inhibits the replication of human coronavirus OC43 (HCoV-OC43), a member of beta-coronaviruses that infects humans. Briefly, HUVEC cells were infected with HCoV-OC43 at an MOI of ∼0.1and treated with K31 at increasing concentrations for 24 h post infection, as mentioned in Experimental procedures. Virus replication was examined by quantification of viral genomic RNA as mentioned above. It is evident from Figure 6, *B* and *C* that similar to FCoV, K31 inhibited HCoV-OC43 replication in cell culture with an EC_50_ of 1 ± 0.1 μM and selective index of 89 (Table 3).

**Table 3:** Chemical inhibition of FCoV and HCoV-OC43 in cell culture

Virus	Chemical compound	EC_50_ (μM)	CC_50_ (μM)	SI
FCoV	K31	1.0 ± 0.2	115 ± 5.0	∼115
FCoV	GS441524	1.1 ± 0.06	>100^a^	>100
FCoV	GC376	0.06 ± 0.003	>150^b^	>2.5 × 10^3^
HCOV-OC43	K31	1.0 ± 0.05	89 ± 6.0^c^	∼89

Selective index (SI) = EC_50_/CC_50_.

a The CC_50_ value of GC376 in CRFK cells was reported in (41).

b The CC_50_ value of GS44154 in CRFK cells was reported in (42).

c The CC_50_ value of K31 in HUVECs was reported in (29).

We next compared the potency of K31 with two well-characterized FCoV inhibitors GS441524 and GC376 in cell culture. Briefly, the CRFK cells were infected with FCoV at an MOI of ∼0.1 as mentioned above and treated with increasing concentrations of GS441524 and GC376 for 24 h post infection. The concentration range of these inhibitors was chosen based on their reported CC_50_ values in CRFK cells (41, 42). Virus replication was examined by real-time PCR, as mentioned above. It is evident from Figure 6, *E*–*H* that GS441524 inhibited FCoV replication with similar potency as K31 (Table 3). However, the potency of GC376 was significantly higher compared with K31 and GS441524, evident from lower EC_50_ value (∼0.06 μM) and higher selective index of ∼2.5 × 10^3^.

### Confirmation of viral inhibition by plaque assay

**Figure 7:**
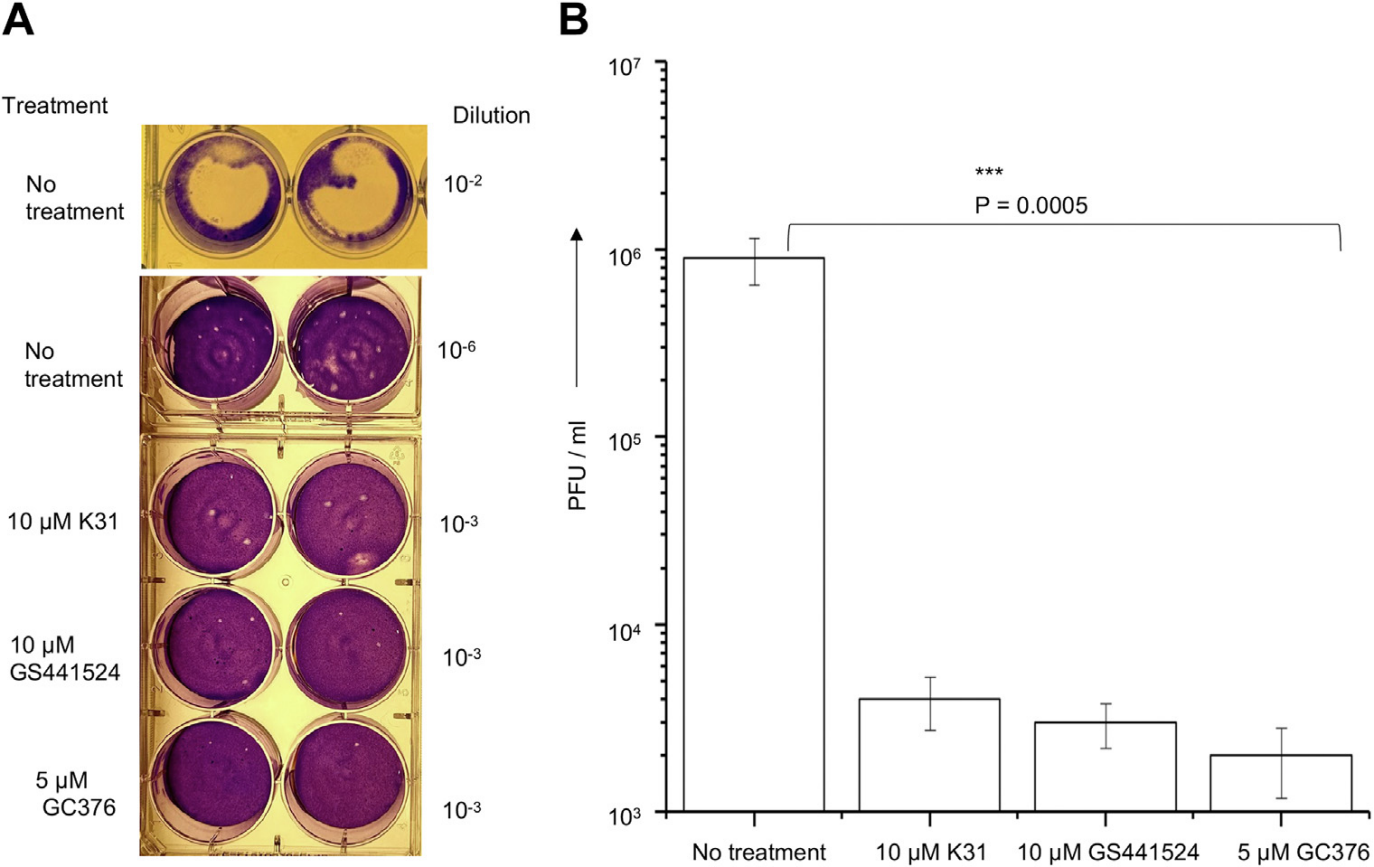
**Plaque assay to examine the viral titers.***A*, CRFK cells infected with FCoV were treated with K31 (10 μM), GS441524 (10 μM), or GC376 (5 μM) or left untreated as control. The medium from infected cells was harvested 24 h post infection and applied to CRFK cells following a 100-fold dilution series. After Oxoid agar-medium overlay the plates were incubated for 3 days in CO_2_ incubator before the cells were fixed with 3.7% formaldehyde and stained with 0.1% crystal violet. Shown is the representative example of the plaque at different dilutions of the harvested medium. Titers were determined by plaque counts and are presented as plaque forming units (PFU)/ml in (*B*).

We next used plaque assay to further confirm the impact of K31, GS441524, and GC376 upon the replication of FCoV in cell culture. CRFK cells at 90% confluency in six-well plates were infected with FCoV at an MOI of 0.1. After absorption for 90 min, cells were washed and incubated with complete medium containing K31 (10 μM), GS441524 (10 μM), or GC376 (5 μM) at 37 °C in CO_2_ incubator for 24 h. Virus released in the medium was quantified by plaque assay, as mentioned in Experimental procedures. Serial dilution of the medium showed well-resolved plaques 3 days post infection. The medium from untreated cells contained high viral load, evident from dramatic cytopathic effects and clearance of cells from the plate at 100-fold dilution of the medium (Fig. 7*A*). The plaque assay revealed that medium from untreated cells contained FCoV at a concentration of ∼10^6^ PFU/ml (Fig. 7*B*). The medium from cells treated with K31, GS441524, and GC376 contained FCoV at a concentration of ∼10^3^ PFU/ml, demonstrating at least 1000-fold reduction in the release of infectious virus particles. The effect was more pronounced by GC376, consistent with similar observation from the real-time PCR approach (Fig. 6). It is evident from Table 3, Figures 6 and 7 that K31 has similar potency as GS441524.

### K31 inhibits the postentry steps of FCoV replication cycle

**Figure 8:**
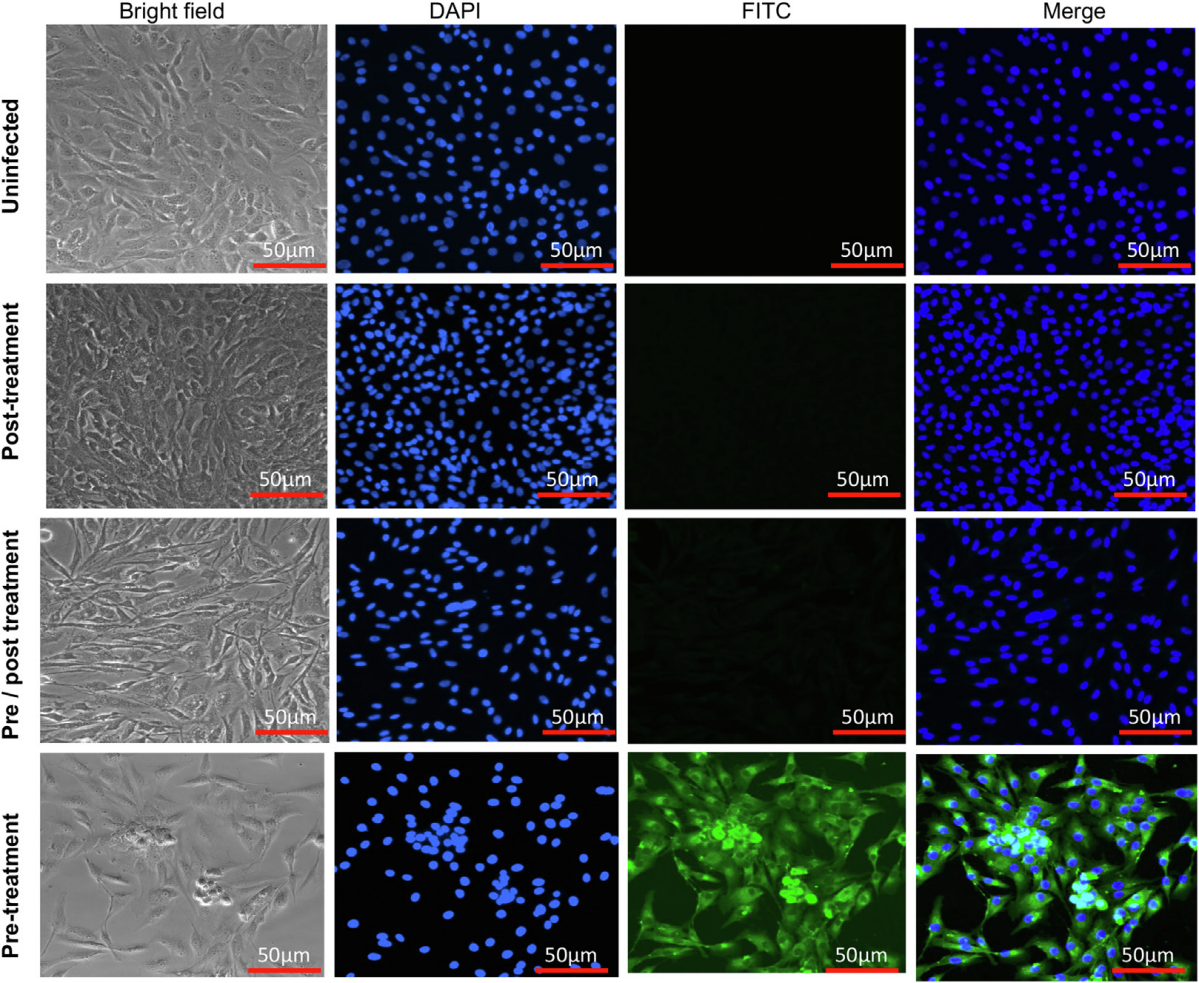
**Mode of action for K31.** CRFK cells seeded in 12-well plates were infected with FCoV (MOI of ∼0.1) and treated with 10 μM K31. The K31 was delivered to cells by pretreatment, posttreatment, or pre/posttreatment, as discussed in the Results. Virus replication was monitored 24 h post infection by the visualization of N using immunofluorescence staining.

To dissect the mode of action, K31 was further examined for virus inhibition using three different approaches as reported (29). Briefly, a fixed concentration of K31 and FCoV inoculum were simultaneously added to cells and incubated for 1 h. Cells were washed and further incubated with fresh medium lacking the inhibitor (pretreatment). The effect exerted on virus replication in this approach would be mostly due to the interference in the entry step of virus replication. Second, the virus inoculum was first incubated with cells in the absence of inhibitor to allow the virus entry to the host cell in the absence of inhibitor. Cells were washed to remove the unattached virus and were further incubated with fresh medium containing the inhibitor. The effect observed on virus replication using this approach (post treatment) would be mostly due to interference in the postentry steps of virus replication. Third, K31 and virus inoculum were simultaneously added to CRFK cells in culture and incubated for 1 h. Cells were washed and further incubated with fresh medium containing K31. Using this approach (pre/post treatment) K31 will perturb either virus entry or postentry steps of virus replication or both. Virus replication in infected cells was monitored 24 h post infection by the immunofluorescence staining, as mentioned in Experimental procedures. It is evident from Figure 8 that K31 had no impact upon FCoV replication when delivered by the pretreatment approach, suggesting that K31 does not interfere in the entry steps of FCoV replication. K31 likely exerts its antiviral affects by perturbing the postentry steps of FCoV replication cycle, evident from significant virus inhibition when K31 was delivered by both posttreatment and pre/posttreatment approaches (Fig. 8).

### K31 dismantles the structural integrity of FCoV ribonucleocapsids in infected cells

**Figure 9:**
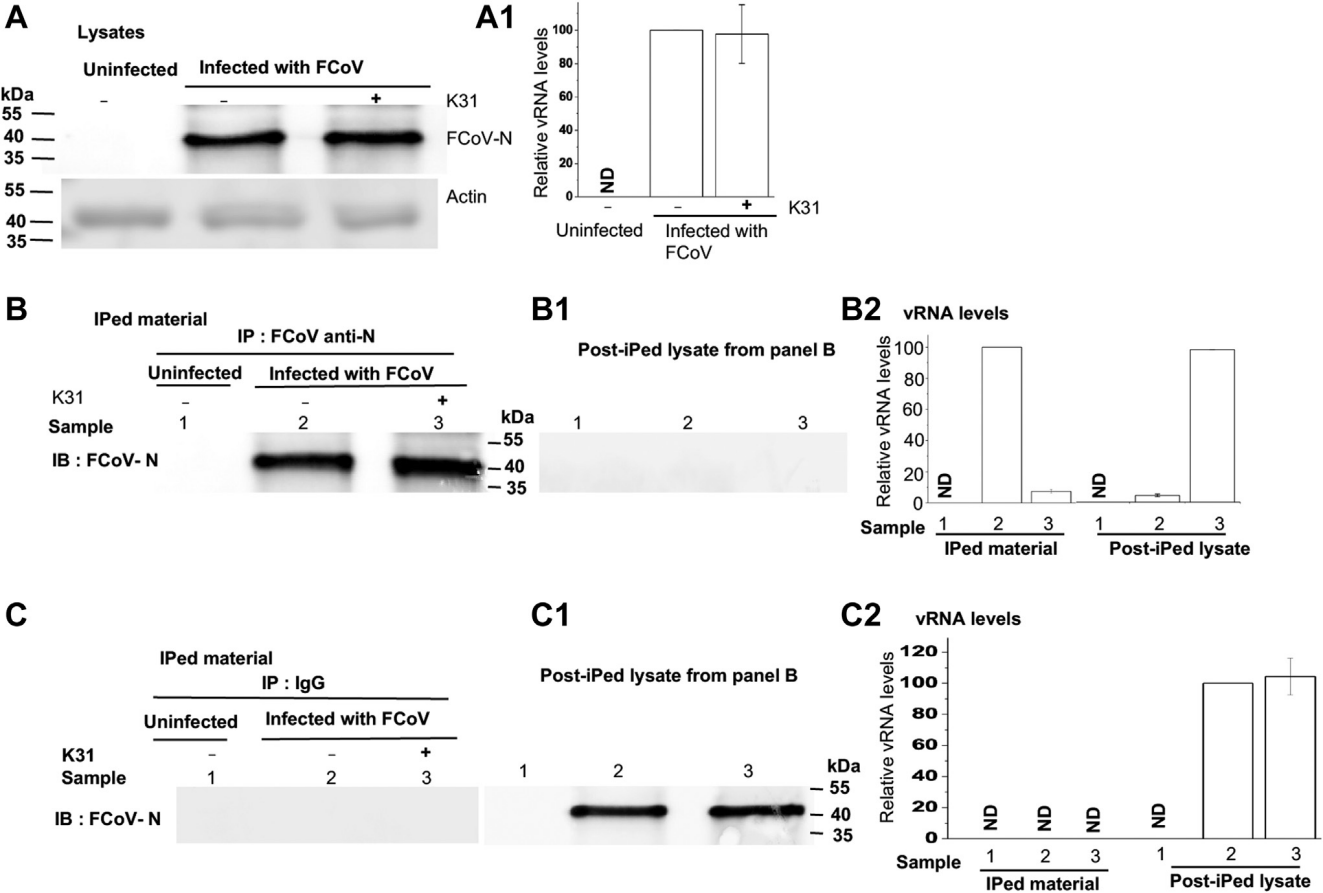
**K31 inhibits vRNA–N interaction in virus-infected cells.***A*, CRFK cells were grown in three 10-cm dishes. Two of the dishes were infected with FCoV (MOI of ∼0.1). One of the infected dishes was treated with 30 μM K31 2 h before harvesting. Cells from all three dishes were lysed 24 h post infection. A volume of 20 μl of the lysate was separated on SDS-PAGE gel and examined by Western blot analysis using mouse anti-N monoclonal antibody. *A*1, total RNA was purified from 50 μl of the lysate, and FCoV genomic RNA was quantified by real-time PCR. *B* and *B*1, the lysate from *A* was immunoprecipitated by mouse anti-N monoclonal antibody. The lysate after immunoprecipitation referred to as “post-iPed lysate” was saved. The immunoprecipitated material (*B*) and post-iPed lysate (*B*1) were examined by Western blot analysis using guinea pig anti-FIP serum as primary antibody and anti–guinea pig secondary antibody. *B*2, total RNA was purified from both iPed material and post-iPed lysate, and FIP vRNA was quantified by real-time PCR. *C* and *C*1, the lysate from A was immunoprecipitated by mouse IgG. The immunoprecipitated material (*C*) and post-iPed lysate (*C*1) were examined by Western blot analysis as mentioned in *B* and *B*1. *C*2, total RNA was purified from both iPed material and post-iPed lysate, and FIP vRNA was quantified by real-time PCR.

To further understand the mechanism of action at the postentry step of virus replication cycle, we asked whether K31 impacted the structural integrity of viral ribonucleocapsids in infected cells, especially due to observed inhibition of N–RNA interaction *in vitro* (Fig. 2). To test this hypothesis, CRFK cells were seeded in 10-cm dishes, followed by FCoV infection at an MOI of 0.1. Cells were lysed 24 h post infection. One of the infected dishes was treated with 30 μM K31 2 h before lysis. Cell lysates were examined for the presence of FCoV N by Western blot analysis (Fig. 9*A*) and the quantification of viral genomic RNA by real-time PCR analysis (Fig. 9A1). It is clear from Figure 9*A* and *A*1 that K31 treatment for 2 h before lysis did not have any noticeable impact upon virus replication, evident from equal N and vRNA levels in both K31-treated and untreated cells.

Since N in association with viral genomic RNA forms stable ribonucleocapsids in virus-infected cells, they copurify together demonstrating their structural integrity. To determine whether K31 treatment impacted the integrity of ribonucleocapsids in FCoV-infected cells, the cell lysates were immunoprecipitated with anti-N monoclonal antibody. Both the immunoprecipitated material and the lysate after immunoprecipitation, referred as post-iped lysate, were tested for the presence of N and FCoV genomic RNA using Western blot and real-time PCR approaches, respectively. The monoclonal antibody equally immunoprecipitated the N from both K31-treated and untreated cell lysates (Fig. 9*B*). Western blot analysis revealed that N was detected only in the immunoprecipitated material and not in the post-iped lysate, demonstrating an effective immunoprecipitation (compare *B* and *B*1 in Fig. 9). The real-time PCR analysis of both the immunoprecipitated material and post-iped lysate revealed that vRAN copurified with N in untreated CRFK cells. This is evident by the presence of both N and vRNA in immunoprecipitated material and not in post-iped lysate from untreated CRFK cells (compare sample two in Fig. 9*B*, *B*1 and *B*2). This demonstrates the structural integrity of FCoV ribonucleocapsids in untreated CRFP cells. Interestingly, the vRNA in K31-treated CRFK cells did not copurify with N, as the majority of it was present in the post-iped lysate (compare samples 3 in Fig. 9*B*, *B*1 and *B*2). This clearly demonstrates that K31 dismantled the ribonucleocapsids in FIPV-infected cells, enabling the immunoprecipitation of N independent of the vRNA. This interesting observation has shed light on the molecular mechanism by which K31 inhibits FCoV replication in cells. Dismantling the structural integrity of ribonucleocapsids by K31 will prevent them to serve as efficient template for both transcription and replication of the viral genome. Figure 9*C* demonstrates that IgG (control) did not immunoprecipitate the N, evident from the presence of both N and vRNA in post-iped lysate and not in the immunoprecipitated material.

## Discussion

The huge loss of world economy and staggering death toll in COVID-19 pandemic has made it clear that coronaviruses remain a major threat to the global health and thus the development of anticoronavirus therapeutics remains a higher priority worldwide. While a majority of anticoronavirus drug development efforts target RdRp (43, 44), spike protein (45), envelop protein (46), 3CL^pro^ (47, 48) and M^pro^ (49, 50), the nucleocapsid protein (N), an important therapeutic target has not been much explored. The antiviral activity of K31 targeting the FCoV N demonstrates that N is an important druggable target for therapeutic intervention of coronaviruses. In addition, the highly conserved structural architecture of coronavirus N might lead to the development of broad-spectrum anticoronavirus therapeutics. Although much research has been carried out on FIP, it still remains one of the most prevalent and fatal diseases of cats. FIP is the leading cause of death among young cats under 2 years of age. FIP is estimated to kill 1 in 100 to 300 cats worldwide (51). FIP also affects endangered exotic cats in zoos, such as jaguars and cheetahs (52). The vaccines have proven ineffective, and the treatment is only palliative. The nucleoside analogue GS-441524 has shown promising results in preventing FIP (8). However, to our knowledge the analogue has not been approved yet by the US Food and Drug Administration for clinical use in treating FIP. Clinically, the majority of FIP is caused by serotype 1 viruses and not by the serotype two against which K31 was tested in this article. Since N is highly conserved in coronaviruses, it is highly likely that K31 will target all serotypes of FCoV including the serotype I.

Our results demonstrate that K31 binds to the FCoV N with a dissociation constant (K_d_) of ∼246 to 340 nM. The binding of K31 induces a conformational change in N, evident from significant reduction in the number of binding sites for the fluorescent probe bis-ANS. K31 noncompetitively inhibited the interaction between purified N and synthetic vRNA 5′ NCR *in vitro* with an inhibition constant (IC_50_) of ∼0.85 μM (Fig. 2). K31 was well tolerated by CRFK cells in culture, evident from CC_50_ of ∼115 μM. Previous studies have also shown similar CC_50_ values of K31 in HUVECs, HeLa, and HEK293T cells (29). K31 inhibited FCoV replication in CRFK cells with an EC_50_ of ∼1 ± 0.1 μM, generating a selective index of 115. K31also inhibited the replication of HCOV-OC43 in HUVECs with a selective index of ∼89. Similar potency was observed with GS441524 (Table 3). However, GC376 showed better efficacy compared with K31 and GS441524 by both plaque assay (Fig. 7) and real-time PCR approach (Fig. 6 and Table 3). The results from Figure 8 clearly demonstrate that K31 does not interfere in the virus entry but inhibits the postentry steps of virus replication. The well-characterized role of N in virus replication is the specific recognition and encapsidation of viral genomic RNA into viral ribonucleocapsids, which then serve as templates for transcription and replication of the viral genome. N also plays diverse roles in transcription and replication of the viral genome in conjunction with replication complex, virus assembly and budding (53), cell cycle regulation, interference in immune defense, host translation shutoff, and template switching during transcription/replication (23, 24). To further gauge insight into the mechanism of action of K31, the interesting immunoprecipitation experiment in Figure 9 demonstrated that K31 treatment disrupted the structural integrity of viral ribonucleocapsids. Interruption in such a critical step of virus replication would have a catastrophic impact upon virus replication, which is evident from the dramatic inhibition of FCoV replication by an overnight treatment of infected cells with a single dose of 10 μM K31 (Figs. 5 and 7). Interruption of K31 in other important functions of N during the course of virus replication cannot be ruled out.

These results hold promise for further development of K31 as an anticoronavirus therapeutic, especially for the treatment of FIP. Limited proteolytic digestion in conjunction with mass spectrometry will be used in future to map the regions in N that undergo structural changes upon binding to K31. A cocrystal structure of the N–K31 complex will provide crucial insights for further modification of K31 to generate new derivatives that might have high target binding affinity and improved anti-FCoV efficacy. In addition, K31 needs to be tested for antiviral activity in infected cats to examine its efficacy in animal model. The inhibition of HCoV-OC43, a beta-coronavirus, by K31 (Fig. 6) demonstrates its broad-spectrum anticoronavirus activity. In addition, both K31 and GS441524, a widely used drug for the treatment of FIP, showed similar anti-FCoV efficacy (Table 3) in cell culture. A combination therapy using K31, GC376, and GS441524 specific to three diverse therapeutic targets, nucleocapsid protein, 3CL^pro^, and replication complex, respectively, might improve the prognosis of FIP in cats.

## Experimental procedures

### Cells and other reagents

Polyclonal Anti-Feline Infectious Peritonitis Virus (FIPV), 79-1146 (antiserum, Guinea Pig) (Cat # NR-2518) and Alphacoronavirus 1, 79-1146, formerly Feline Infectious Peritonitis Virus (FIPV) (Cat # 43287) were from BEI RESOURCES. CRFK cells were from the laboratory of Dr Yvonne Drechsler (Western University of Health Sciences). The CRFK cells were maintained in Dulbecco's modified Eagle's medium (DMEM) containing 10% fetal bovine serum (FBS) and penicillin–streptomycin (100 μg/ml) in a CO_2_ incubator. Anti-FIP N mouse monoclonal antibody (Cat # SM1870P) was from Origene. Anti-guineapig (Cat #: 926-68077) and anti-mouse (Cat #: 926-68072) secondary antibodies were from LI-COR. The PCR reagents were from GenScript. The reagents for real-time PCR were from Applied Biosystems. All other reagents were from Sigma. HisTrap columns were from Cytiva (Cat # 17528601), High Precision Streptavidin (SAX) biosensors were from ForteBio (Cat # 18-0037) and Rosetta(DE3) Competent Cells were from Millipore Sigma (Cat # 70954)..

### Cloning, expression, and purification of FCoV N

The gene encoding the FCoV N (GenBank: AY994055.1) was codon optimized for bacterial expression and synthesized by GenScript. The sequence was incorporated in pET-30a(+) backbone between NdeI and HindIII restriction sites for the expression of C-terminally His-tagged N. The Rosetta (DE3) *E. coli* cells transformed with the resulting plasmid were induced with 1 mM IPTG upon entering into exponential growth phase (*A*_600_ 0.4). Cells were allowed to grow overnight at 16 °C and were harvested by centrifugation at 3000 rpm for 30 min at room temperature. Cells were resuspended in lysis buffer (50 mM Tris-HCl, pH 7.4, 150 mM NaCl, 2 mM dithiothreitol [DTT], 1% Triton-X100, 5 mM CHAPS, 0.1 mM phenyl methyl sulfonyl fluoride [PMSF]), followed by sonication on ice and clearance of the lysate by centrifugation at 4500*g* for 20 min. N was purified from clear lysates using AKTA pure protein purification system (GE Healthcare), as reported (30).

### T7 transcription for the synthesis of FIP vRNA 5′ noncoding region

A single-stranded DNA segment encoding 36 nucleotides of the 5′NC of FCoV genomic RNA, flanked by T7 promoter, was synthesized by IDT. The resulting DNA segment was PCR amplified in a PCR reaction using two opposing primers. The PCR product was gel purified and used as template in an *in vitro* T7 transcription reaction. RNA synthesis was carried out using the T7 RiboMax kit (Promega), following the manufacturer’s instructions. The RNA was biotinylated or radiolabeled during synthesis as reported (31, 32, 33).

### RNA filter binding analysis

Interaction of bacterially expressed and purified FCoV N with the RNA of interest (Fig. 1 and Table 1) was studied by filter binding assay, as reported (33, 37). Briefly, the 36 nucleotides from the 5′ terminus of viral genomic RNA were synthesized *in vitro* using T7 RNA polymerase and radiolabeled with [α^32^P] GTP during synthesis, as mentioned above. All binding reactions were carried out in RNA binding buffer (40 mM Tris-HCl [pH 7.4], 80 mM NaCl, 20 mM KCl, 1 mM DTT) at a fixed concentration of synthetic 5′ NCR with increasing concentrations of N. Reaction mixtures were incubated at room temperature for 30 to 45 min and filtered through nitrocellulose membranes under vacuum. Filters were washed with 10 ml of RNA binding buffer and dried. The amount of radiolabeled RNA retained on the filter at each input concentration of N was measured by quantifying the radioactive signal, using a scintillation counter. The background signal from nonspecific binding of RNA to the filter was subtracted from each data point. A binding profile was generated by plotting the radioactive signal along *y*-axis and N concentration along *x*-axis. The percentage of bound RNA at each input concentration of N was calculated using Equation 1.

(1) $$Percentage\;of\;bound\;RNA=\Delta R\;/\;\Delta R_{\max}*100$$

where ΔR is the change in radioactive signal at each addition of N. ΔR_max_ is the same parameter when the RNA is totally bound to the N. Double reciprocal plot (1/*ΔR versus* 1*/C*_*p*_, (37)) was used to calculate the value of *ΔR*_*max*_, using Equation 2. C_p_ is the input N concentration.

(2) $$1/\Delta R=1/\Delta R_{\max}{+K}_{d}/\left(\Delta R_{\max}{*C}_{p}\right)$$

Since Equation 2 is valid for the calculation of *ΔR*_*max*_ under the conditions where Cp >> initial concentration of RNA (54), only the data points corresponding to the saturation phase of the binding profile were fitted to Equation 2 for the calculation of *ΔR*_*max*_. Alternatively, *ΔR*_*max*_ was calculated by simply averaging the radioactive signal of saturating data points. The percentage of bound RNA obtained from Equation 1 was plotted *versus* input N concentration, and the resulting data points were fit to a dose–response equation using the program Origin six (Microcal). The apparent dissociation constant (*Kd*) corresponded to the concentration of N protein required to obtain the half-saturation in the fitted binding curve, assuming that the complex formation obeys a simple bimolecular equilibrium.

### Biolayer interferometry

Biolayer interferometry was used to examine the binding affinity of purified N with the synthetic biotinylated 5′ NCR sequence of viral genomic RNA, using the BLITZ system (ForteBio Inc), as reported (31, 32, 33). Briefly, the synthetic biotin-5′ NCR was loaded onto high-precision streptavidin biosensors (cat # 18-5019, Forte Bio Inc), as reported (55). All reactions were carried out at room temperature in RNA binding buffer (20 mM Tris-HCl, pH 7.4, 80 mM NaCl, 20 mM KCl, and 1 mM DTT). After mounting the RNA, the biosensors were equilibrated in RNA binding buffer and then dipped in the purified N solution for the measurement of association kinetics. The reaction cycles were as follows: initial base line for 30 s, loading of biotinylated RNA on streptavidin biosensors for 200 s, base line for 30 s, association of protein with the RNA for 400 s, followed by dissociation phase of 400 s. The kinetic parameters K_ass_ (association rate constant), K_dis_ (dissociation rate constant) and the binding affinities (K_d_ = K_dis_/K_ass_) were calculated with the help of inbuilt data analysis software (BLItZ Pro), as reported (31, 32, 33).

The interaction of K31 with N was also studied using the BLI, as reported (29). Briefly, the purified His-tagged N in RNA binding buffer was immobilized on high-precision Ni-NTA biosensors (cat # 18-5101, Forte Bio Inc), as reported (29). The biosensors were equilibrated with RNA binding buffer and then dipped in K31 solutions, containing K31 at different concentrations in RNA binding buffer to monitor the association kinetics. The reaction cycles and calculation of kinetic parameters were the same as mentioned above.

### Inhibition of N–K31 interaction using BLI

To determine the impact of K31 upon the interaction of N with the vRNA 5′ NCR, the experiment was carried out similar to N–NCR interaction mentioned above except that N solution was replaced with the solution of N–K31 complex. Briefly, after mounting the biotinylated 5′ NCR on high-precision streptavidin biosensors, the biosensors were dipped in a solution containing N–K31 complexes. The complexes were formed by incubating a fixed concentration of purified N (120 nM) with increasing concentrations of K31 in RNA binding buffer at room temperature for 45 min. The association and dissociation kinetic phases of the resulting N–K31 complexes with the biotinylated 5′ NCR, immobilized on streptavidin biosensor, were recorded. The binding signal at the saturation time point (595 s) was recorded for each input K31 concentration. The recorded binding signal was normalized relative to the control signal lacking K31 for the calculation of percent N bound to 5′ NCR at each input K31 concentration. The percentage of N bound at each input concentration of K31 was then plotted, and the data points were fit to a dose–response equation using nonlinear least-squares analysis (GraphPad Prism). The IC_50_ value represented the concentration of K31 at which the binding signal was reduced by 50%.

### Inhibition of N–K31 interaction using filter binding assay

To confirm that K31 inhibited the interaction of N with the vRNA 5′ NCR, the 5′ NCR sequence was synthesized by T7 RNA polymerase and radiolabeled with [α^32^P] GTP as mentioned above. As previously reported (29), a complex between N and 5′ NCR was formed by incubating a fixed concentration of N (300 nM) with a fixed concentration of 5′ NCR (∼20,000 cpm) in RNA binding buffer at room temperature for 30 min. The resulting complex was incubated with increasing concentrations of K31 at room temperature for additional 30 min, followed by filtration of the resulting mixture through nitrocellulose filter under vacuum. Filters were washed with 10 ml of RNA binding buffer and dried. The amount of N–5′ NCR complex retained on the filter at each input concentration of K31 was measured by quantifying the radioactive signal, using a scintillation counter. The decreasing radioactive signal at each input concentration of K31was normalized related to the control lacking the K31 and plotted *versus* K31concentration to generate the inhibition plot. The data points were fit to a dose–response equation using nonlinear least square analysis as reported (29). The IC_50_ value represented the concentration of K31 at which 50% of the N–5′ NCR complex was dissociated, calculated from 50% reduction in the normalized radioactive signal in the inhibition plot, as reported (29).

To define the mechanism of inhibition, N–5′ NCR complexes were formed by incubating a fixed concentration of radiolabeled 5′ NCR (∼6000 cpm) with increasing concentrations of N ranging from 0 to 200 nM in RNA binding buffer at room temperature for 30 min. The resulting complexes were further incubated with K31 at four different concentrations (0.0–0.35 μM) at room temperature for additional 30 min, followed by filtration of the mixture through nitrocellulose filter. The radioactive signal retained on the washed filters was subtracted from the background signal at zero N concentration to calculate ΔR. The resulting ΔR values were plotted *versus* input N concentration to generate four binding profiles corresponding to four K31 concentrations (Fig. 2*E*). The Lineweaver–Burk plots (Fig. 2*F*) were generated by plotting 1/ΔR *versus* 1/[N], and the data points were fit to straight line (Fig. 2*F*). The slops from four Lineweaver–Burk plots corresponding to four K31 concentrations (Fig. 2*F*) were plotted *versus* input K31 concentrations to generate a secondary plot (Fig. 2*G*). The data points in Figure 2*G* were fit to a straight line. The intercept on *x*-axis corresponds to -Ki, the dissociation constant for N–K31 interaction.

### Cytotoxicity

The impact of K31 upon the viability of CRFK cells was determined using CellTiter-Glo luminescent assay reagent according to the manufacturer’s instructions (Promega), as reported (29). Briefly, 10,000 CRFK cells were seeded in each well of a 96-well plate and incubated for 48 h in 100 μl medium containing increasing concentrations of K31. Control wells containing medium with increasing concentrations of K31 without cells were also prepared. The plate was equilibrated at room temperature for 30 min, followed by the addition of 100 μl of the Cell titer-Glo reagent. The Cell titer-Glo reagent was prepared following manufacturer’s instructions. The plate was incubated for 2 min on an orbital shaker at room temperature to induce cell lysis. The plate was incubated for additional 10 min at room temperature to stabilize the luminescent signal. The luminescence was recorded on a plate reader (PROMEGA GloMax Explorer). The luminescence signal for each sample was subtracted from the corresponding negative control. Since the small molecule inhibitors were dissolved in 1% DMSO, the cell viability in each well was normalized relative to viability observed at 1% DMSO without K31.

### Propagation of FCoV and human coronavirus OC43

Feline infectious Peritonitis virus (strain 79-1146), formally known as FCoV, was from BEI Resources (Cat #: NR-43287). The virus was propagated in CRFK cells by infecting 80% confluent monolayers of CRFK cells with FCoV at an MOI of 0.01. Virus-infected cells were cultured for 7 days in viral growth medium (Opti-MEM from Gibco, Cat # 51985-034, containing 2.5% FBS). The viral growth medium containing budded virus particles was harvested, cleared by low-speed centrifugation, and stored in 1-ml aliquots at −80 °C in MEM containing 10% FBS. HCoV-OC43 was from ATCC (VR-1558). The virus was propagated in HCT-8 cells from ATCC (CCL-244). Briefly, HCT-8 cells at a confluency of 80 to 90% were incubated with HCoV-OC43 at an MOI of 0.01 in serum-free Opti-MEM medium at 33 °C inside CO_2_ incubator for 2 h with continuous swirling every 15 min. Unabsorbed virus was removed by washing the cells once with complete medium (Opti-MEM medium containing 10% FBS). Cells were incubated with complete medium for 7 days, and viral growth medium containing budded virions was harvested as mentioned above.

### Replication assays for FCoV and HCoV-OC43

Impact of K31 upon FCoV replication was assayed in CRFK cells. Briefly, CRFK cells seeded in 24-well plates were incubated with FCoV at an MOI of ∼0.1 in Opti-MEM growth medium lacking FBS for 1 h with brief shaking every 15 min. The virus inoculum was removed and replaced with fresh Opti-MEM growth medium containing increasing concentrations of K31, GS441524 (TargetMol, Cat # T7222), or GC376 (TargetMol, Cat # T5188). Since these inhibitors are dissolved and stocked in 100% DMSO, the final concentration of DMSO in Opti-MEM growth medium was 0.1% after the addition of K31. Thus, the growth medium of FCoV-infected CRFK cells lacking the inhibitor was also supplemented with 0.1% DMSO as control. Cells were harvested 24 h post infection, and total RNA was extracted by RNA purification kit (Zymo, Cat # R1055), following the manufacturer’s instructions. Virus replication was monitored by quantitative estimation of viral genomic RNA by real-time PCR using the relative quantification method, as reported (29). The primers used for the quantification of FIP viral RNA were F-primer: 5′ GATACAATTGTAGCTGTGCTTCAAA and R primer: 5′ GTTACCATTGGCAACGAGATCACTA. The primers used for the quantification of β-actin as internal control were F-primer: 5′TCGCCGACAGGATGCAGAAG and R-primer: 5′ AGGTGGACAGCGAGGCCAGG. The same approach was used to examine the impact of K31 upon HCoV-OC43 replication. Briefly, HUVECs in six-well plates were infected with HCoV-OC43 at an MOI of ∼0.1, followed by incubation with increasing concentrations of K31 for 24 h post infection. RNA purification and quantification of viral genomic RNA was carried out by real-time PCR, as mentioned above. The primers used were F-primer: CCCAAGCAAACTGCTACCTCTCAG and R-primer: GTAGACTCCGTCAATATCGGTGCC.

### Plaque assay

Plaque assay was used to quantify replication-competent FCoV particles in the medium harvested from CRFK cells that were infected with FCoV and treated with K31, GS441524, or GC376, as discussed in Results section. Briefly, the confluent CRFK cells in six-well plates were infected with 100-fold dilution series of the test medium and the plates were incubated for 90 min at 37 °C in CO_2_ incubator with constant swirling every 15 min. The dilution series of the test samples was generated using DMEM lacking FBS. After virus absorption, cells were overlayed with complete DMEM containing 1% FBS and penicillin–streptomycin (100 μg/ml) and 0.5% Oxoid agar (Fisher, #LP0028), followed by incubation in CO_2_ incubator for 3 days. Cells were fixed with 3.7% formaldehyde in 1× PBS solution for 30 min. Viral plaques were visualized by staining with 0.1% crystal violet for 30 min. It must be noted that plaques were visible with high clarity when Oxoid agar was used.

### Immunofluorescence staining

Briefly, CRFK cells seeded in 24-well plates were infected with FCoV at an MOI of ∼0.1. The virus was allowed to replicate in the presence or absence of K31 or DMSO as negative control. Infected cells were fixed 24 h post infection for 10 min at room temperature using ice-cold 100% methanol. Cells were washed three times with 1× PBS and permeabilized with 0.1% Triton X-100 in 1× PBS at room temperature for 10 min. Cells were blocked with 1% bovine serum albumin in 1× PBS at room temperature for 30 min. Cells were incubated with polyclonal guinea pig anti-FIP serum (BEI Resources, Cat # NR-2518) at 1:200 dilution in 1× PBS containing 1% bovine serum albumin for 1.5 h at room temperature. Cells were washed 3 times with 1× PBS and incubated for 1 h with FITC-conjugated rabbit anti–guinea pig IgG (Cat #: A18884, Fisher Scientific). Cells were washed 3 times with 1× PBS and incubated with DAPI (Thermo Fisher, Cat #: 62248) at 1:1000 dilution in 1× PBS at room temperature for 5 min. Cells were again washed three times with 1× PBS and visualized under fluorescence microscope. Alternatively, mouse monoclonal anti-FIP N antibody (Origene, Cat #: SM1870P) along with FITC-conjugated anti-mouse secondary antibody can be used, which gives similar results.

### Bis-ANS binding

Fluorescence studies of the hydrophobic fluorophore bis-ANS (Sigma, Cat # 65664-81-5) were carried out in a Shimadzu spectrofluorometer (RF-5301PC) as reported (29). The fluorophore was dissolved in DMSO to a final concentration of 10 mM. The fluorophore was excited at 399 nm, and the emission spectrum was recorded from 420 to 600 nm. To a fixed concentration of N (850 nM) in the reaction buffer (20 mM Tris-HCl pH 8.0, 100 mM NaCl and 3% DMSO), small aliquots of bis-ANS were added from a higher-concentration stock and fluorescence intensity at 485 nm was recorded at each input concentration of bis-ANS. Similarly, N was first incubated with K31 at room temperature for 45 min, followed by the addition of small aliquots of bis-ANS from a higher-concentration stock and fluorescence intensity at 485 nm was recorded. To determine the change in fluorescence signal of bis-ANS due to binding with N, the fluorescence signal of free bis-ANS in the reaction buffer without N was subtracted. The subtracted fluorescence signal was plotted *versus* each input concentration of bis-ANS.

## Data availability

All the data have been included in the article.

## Conflict of interest

The authors declare that they have no conflicts of interest with the contents of this article.

## References

[1] Sparkes A.H., Gruffydd-Jones T.J., Harbour D.A. (1992). Feline coronavirus antibodies in UK cats. Vet. Rec..

[2] Addie D.D., Jarrett O. (1992). A study of naturally occurring feline coronavirus infections in kittens. Vet. Rec..

[3] Vennema H., Poland A., Foley J., Pedersen N.C. (1998). Feline infectious peritonitis viruses arise by mutation from endemic feline enteric coronaviruses. Virology.

[4] Poland A.M., Vennema H., Foley J.E., Pedersen N.C. (1996). Two related strains of feline infectious peritonitis virus isolated from immunocompromised cats infected with a feline enteric coronavirus. J. Clin. Microbiol..

[5] Pedersen N.C., Eckstrand C., Liu H., Leutenegger C., Murphy B. (2015). Levels of feline infectious peritonitis virus in blood, effusions, and various tissues and the role of lymphopenia in disease outcome following experimental infection. Vet. Microbiol..

[6] Vuong W., Khan M.B., Fischer C., Arutyunova E., Lamer T., Shields J. (2020). Author Correction: feline coronavirus drug inhibits the main protease of SARS-CoV-2 and blocks virus replication. Nat. Commun..

[7] Vuong W., Khan M.B., Fischer C., Arutyunova E., Lamer T., Shields J. (2020). Feline coronavirus drug inhibits the main protease of SARS-CoV-2 and blocks virus replication. Nat. Commun..

[8] Pedersen N.C., Perron M., Bannasch M., Montgomery E., Murakami E., Liepnieks M. (2019). Efficacy and safety of the nucleoside analog GS-441524 for treatment of cats with naturally occurring feline infectious peritonitis. J. Feline Med. Surg..

[9] Legendre A.M., Kuritz T., Galyon G., Baylor V.M., Heidel R.E. (2017). Polyprenyl immunostimulant treatment of cats with presumptive non-effusive feline infectious peritonitis in a field study. Front. Vet. Sci..

[10] Prajapat M., Sarma P., Shekhar N., Avti P., Sinha S., Kaur H. (2020). Drug targets for corona virus: a systematic review. Indian J. Pharmacol..

[11] Jean A., Quach C., Yung A., Semret M. (2013). Severity and outcome associated with human coronavirus OC43 infections among children. Pediatr. Infect. Dis. J..

[12] To K.K., Sridhar S., Chiu K.H., Hung D.L., Li X., Hung I.F. (2021). Lessons learned 1 year after SARS-CoV-2 emergence leading to COVID-19 pandemic. Emerg. Microbes Infect..

[13] Dye C., Siddell S.G. (2007). Genomic RNA sequence of feline coronavirus strain FCoV C1Je. J. Feline Med. Surg..

[14] Fan H., Ooi A., Tan Y.W., Wang S., Fang S., Liu D.X. (2005). The nucleocapsid protein of coronavirus infectious bronchitis virus: crystal structure of its N-terminal domain and multimerization properties. Structure.

[15] Yu I.M., Oldham M.L., Zhang J., Chen J. (2006). Crystal structure of the severe acute respiratory syndrome (SARS) coronavirus nucleocapsid protein dimerization domain reveals evolutionary linkage between corona- and arteriviridae. J. Biol. Chem..

[16] Yu I.M., Gustafson C.L., Diao J., Burgner J.W., Li Z., Zhang J. (2005). Recombinant severe acute respiratory syndrome (SARS) coronavirus nucleocapsid protein forms a dimer through its C-terminal domain. J. Biol. Chem..

[17] Chen C.Y., Chang C.K., Chang Y.W., Sue S.C., Bai H.I., Riang L. (2007). Structure of the SARS coronavirus nucleocapsid protein RNA-binding dimerization domain suggests a mechanism for helical packaging of viral RNA. J. Mol. Biol..

[18] Baric R.S., Nelson G.W., Fleming J.O., Deans R.J., Keck J.G., Casteel N. (1988). Interactions between coronavirus nucleocapsid protein and viral RNAs: implications for viral transcription. J. Virol..

[19] Verheije M.H., Hagemeijer M.C., Ulasli M., Reggiori F., Rottier P.J., Masters P.S. (2010). The coronavirus nucleocapsid protein is dynamically associated with the replication-transcription complexes. J. Virol..

[20] Keane S.C., Giedroc D.P. (2013). Solution structure of mouse hepatitis virus (MHV) nsp3a and determinants of the interaction with MHV nucleocapsid (N) protein. J. Virol..

[21] Hurst K.R., Ye R., Goebel S.J., Jayaraman P., Masters P.S. (2010). An interaction between the nucleocapsid protein and a component of the replicase-transcriptase complex is crucial for the infectivity of coronavirus genomic RNA. J. Virol..

[22] Narayanan K., Maeda A., Maeda J., Makino S. (2000). Characterization of the coronavirus M protein and nucleocapsid interaction in infected cells. J. Virol..

[23] Zuniga S., Sola I., Alonso S., Enjuanes L. (2004). Sequence motifs involved in the regulation of discontinuous coronavirus subgenomic RNA synthesis. J. Virol..

[24] Zuniga S., Cruz J.L., Sola I., Mateos-Gomez P.A., Palacio L., Enjuanes L. (2010). Coronavirus nucleocapsid protein facilitates template switching and is required for efficient transcription. J. Virol..

[25] Surjit M., Kumar R., Mishra R.N., Reddy M.K., Chow V.T., Lal S.K. (2005). The severe acute respiratory syndrome coronavirus nucleocapsid protein is phosphorylated and localizes in the cytoplasm by 14-3-3-mediated translocation. J. Virol..

[26] Kopecky-Bromberg S.A., Martinez-Sobrido L., Frieman M., Baric R.A., Palese P. (2007). Severe acute respiratory syndrome coronavirus open reading frame (ORF) 3b, ORF 6, and nucleocapsid proteins function as interferon antagonists. J. Virol..

[27] Zhou B., Liu J., Wang Q., Liu X., Li X., Li P. (2008). The nucleocapsid protein of severe acute respiratory syndrome coronavirus inhibits cell cytokinesis and proliferation by interacting with translation elongation factor 1alpha. J. Virol..

[28] Roy A., Mir M.A. (2017). Development of high-throughput screening assay for antihantaviral therapeutics. SLAS Discov..

[29] Salim N.N., Ganaie S.S., Roy A., Jeeva S., Mir M.A. (2016). Targeting a novel RNA-protein interaction for therapeutic intervention of hantavirus disease. J. Biol. Chem..

[30] Royster A., Mir S., Mir M.A. (2021). A novel approach for the purification of aggregation prone proteins. PLoS One.

[31] Ganaie S.S., Haque A., Cheng E., Bonny T.S., Salim N.N., Mir M.A. (2014). Ribosomal protein S19 binding domain provides insights into hantavirus nucleocapsid protein-mediated translation initiation mechanism. Biochem J.

[32] Jeeva S., Cheng E., Ganaie S.S., Mir M.A. (2017). Crimean-Congo hemorrhagic fever virus nucleocapsid protein augments mRNA translation. J. Virol..

[33] Jeeva S., Pador S., Voss B., Ganaie S.S., Mir M.A. (2017). Crimean-Congo hemorrhagic fever virus nucleocapsid protein has dual RNA binding modes. PLoS One.

[34] Almeida Filho L.C., Tabosa P.M., Hissa D.C., Vasconcelos I.M., Carvalho A.F. (2018). First insights into insecticidal activity against Aedes aegypti and partial biochemical characterization of a novel low molecular mass chymotrypsin-trypsin inhibitor purified from Lonchocarpus sericeus seeds. Pest Manag. Sci..

[35] Shen J., Wang B., Wang S., Chen F., Meng D., Jiang H. (2020). Effects of voriconazole on the pharmacokinetics of vonoprazan in rats. Drug Des. Devel. Ther..

[36] Palmer T. (2011). Enzymes.

[37] Mir M.A., Sheema S., Haseeb A., Haque A. (2010). Hantavirus nucleocapsid protein has distinct m7G Cap- and RNA-binding sites. J. Biol. Chem..

[38] Haque A., Mir M.A. (2010). Interaction of hantavirus nucleocapsid protein with ribosomal protein S19. J. Virol..

[39] Silva J.L., Silveira C.F., Correia Junior A., Pontes L. (1992). Dissociation of a native dimer to a molten globule monomer. Effects of pressure and dilution on the association equilibrium of arc repressor. J. Mol. Biol..

[40] Ferrao-Gonzales A.D., Palmieri L., Valory M., Silva J.L., Lashuel H., Kelly J.W. (2003). Hydration and packing are crucial to amyloidogenesis as revealed by pressure studies on transthyretin variants that either protect or worsen amyloid disease. J. Mol. Biol..

[41] Kim Y., Liu H., Galasiti Kankanamalage A.C., Weerasekara S., Hua D.H., Groutas W.C. (2016). Reversal of the progression of fatal coronavirus infection in cats by a broad-spectrum coronavirus protease inhibitor. PLoS Pathog..

[42] Murphy B.G., Perron M., Murakami E., Bauer K., Park Y., Eckstrand C. (2018). The nucleoside analog GS-441524 strongly inhibits feline infectious peritonitis (FIP) virus in tissue culture and experimental cat infection studies. Vet. Microbiol..

[43] Kabinger F., Stiller C., Schmitzova J., Dienemann C., Kokic G., Hillen H.S. (2021). Mechanism of molnupiravir-induced SARS-CoV-2 mutagenesis. Nat. Struct. Mol. Biol..

[44] Kokic G., Hillen H.S., Tegunov D., Dienemann C., Seitz F., Schmitzova J. (2021). Mechanism of SARS-CoV-2 polymerase stalling by remdesivir. Nat. Commun..

[45] Du L., He Y., Zhou Y., Liu S., Zheng B.J., Jiang S. (2009). The spike protein of SARS-CoV--a target for vaccine and therapeutic development. Nat. Rev. Microbiol..

[46] Pervushin K., Tan E., Parthasarathy K., Lin X., Jiang F.L., Yu D. (2009). Structure and inhibition of the SARS coronavirus envelope protein ion channel. PLoS Pathog..

[47] Jo S., Kim S., Shin D.H., Kim M.S. (2020). Inhibition of SARS-CoV 3CL protease by flavonoids. J. Enzyme Inhib. Med. Chem..

[48] Turlington M., Chun A., Tomar S., Eggler A., Grum-Tokars V., Jacobs J. (2013). Discovery of N-(benzo[1,2,3]triazol-1-yl)-N-(benzyl)acetamido)phenyl) carboxamides as severe acute respiratory syndrome coronavirus (SARS-CoV) 3CLpro inhibitors: identification of ML300 and noncovalent nanomolar inhibitors with an induced-fit binding. Bioorg. Med. Chem. Lett..

[49] Ghahremanpour M.M., Tirado-Rives J., Deshmukh M., Ippolito J.A., Zhang C.H., Cabeza de Vaca I. (2020). Identification of 14 known drugs as inhibitors of the main protease of SARS-CoV-2. ACS Med. Chem. Lett..

[50] Ghahremanpour M.M., Tirado-Rives J., Deshmukh M., Ippolito J.A., Zhang C.H., de Vaca I.C. (2020). Identification of 14 Known Drugs as Inhibitors of the Main Protease of SARS-CoV-2. bioRxiv.

[51] Rohrbach B.W., Legendre A.M., Baldwin C.A., Lein D.H., Reed W.M., Wilson R.B. (2001). Epidemiology of feline infectious peritonitis among cats examined at veterinary medical teaching hospitals. J. Am. Vet. Med. Assoc..

[52] Heeney J.L., Evermann J.F., McKeirnan A.J., Marker-Kraus L., Roelke M.E., Bush M. (1990). Prevalence and implications of feline coronavirus infections of captive and free-ranging cheetahs (Acinonyx jubatus). J. Virol..

[53] Lu S., Ye Q., Singh D., Cao Y., Diedrich J.K., Yates J.R. (2021). The SARS-CoV-2 nucleocapsid phosphoprotein forms mutually exclusive condensates with RNA and the membrane-associated M protein. Nat. Commun..

[54] Mir M.A., Dasgupta D. (2001). Association of the anticancer antibiotic chromomycin A(3) with the nucleosome: role of core histone tail domains in the binding process. Biochemistry.

[55] Jeeva S., Mir S., Velasquez A., Ragan J., Leka A., Wu S. (2019). Crimean-Congo hemorrhagic fever virus nucleocapsid protein harbors distinct RNA-binding sites in the stalk and head domains. J. Biol. Chem..

